# Does Subjective Well-Being Improve Self-Rated Health from Undergraduate Studies to Three Years after Graduation in China?

**DOI:** 10.3390/healthcare11212813

**Published:** 2023-10-24

**Authors:** Xinqiao Liu, Yifan Zhang, Yunfeng Luo

**Affiliations:** 1 School of Education, Tianjin University, Tianjin 300350, China; 2 School of Public Administration, University of Electronic Science and Technology of China, Chengdu 611731, China

**Keywords:** subjective well-being, self-rated health, undergraduate students, longitudinal study, cross-lagged models

## Abstract

The health status of emerging adults is at risk. Although subjective well-being is one of the factors closely associated with health, their longitudinal relationship is not clear among emerging adults. The study aimed to investigate the prospective relationship between self-rated health and subjective well-being in emerging adults. The study collected longitudinal data from a total of 1021 Chinese college students (537 males and 484 females) for five years, including two years in college and three years after graduation. In the baseline survey, the average age of the sample was 21.57 years old. Descriptive statistics indicated that both self-rated health and subjective well-being significantly decreased from the senior year of college to the year after graduation. Correlation analysis revealed that self-rated health and subjective well-being had a significant positive relationship. In the five-wave random intercept cross-lagged panel model, subjective well-being unidirectionally predicted self-rated health. In other words, the subjective well-being in the previous year could positively predict self-rated health in the following year, but the previous self-rated health could not predict subsequent subjective well-being. Given the significance of emerging adulthood to individual development, more attention and care should be dedicated to improving subjective well-being so as to maintain good health and engagement in work.

## 1. Introduction

Emerging adulthood is significant for one’s development, which is also a critical transition for one’s health [1]. Undergraduate students, in particular, are prone to develop bad living habits, such as smoking, drinking, and staying up late, which pose a serious threat to their health [2,3]. For instance, a report in 2019 stated that 53.3% of undergraduate students in the United States had suffered from one or more diseases in the past year [4]. Self-rated health can reflect the individuals’ assessment of their own health status. As a popular health indicator, self-rated health can predict one’s health state with high efficiency and reliability and is widely used in many studies. A survey of college students in France discovered that 20% of the students rated their health as fair or very poor [5]. Among college students in Australia, 36% think their health status is excellent or very good, and 17% think their health status is fair or poor [6]. A study covering Chinese undergraduate students evaluated the health status from three aspects of physical health, mental health, and social health, and the average scores were only 65.03, 66.25, and 66.53 (full score is 100) [7]. Poor health status not only affects students’ normal college life but also may lead to lower subjective well-being and satisfaction with college life [8,9,10]. In addition, as a result of severe work and economic stress, the health status of emerging adults who are at work is equally worrying, especially those who are unemployed [11]. Hasson and Lindfors (2010) noticed that the self-rated health of undergraduate students majoring in nursing presented a steady decline from the last semester of college to the following three years [12]. Similarly, Sokol et al. (2017) discovered that young people’s self-rated health gradually decreased after the age of 21 [13]. Given the prevalence and severity of health problems among undergraduate students, it is urgent to identify factors that influence their health status.

As one of the factors closely associated with health, subjective well-being has been discussed in many studies in recent years [14,15,16]. Well-being can be divided into hedonic well-being and eudemonic well-being [17]. Hedonic well-being emphasizes the acquisition of subjective well-being, while eudemonic well-being refers to the meaning of life and self-realization. Diener et al. (1999) proposed that subjective well-being included life satisfaction, positive emotion, and negative emotion, which was widely accepted in many studies [18]. Since the first World Happiness Report released by the United Nations in 2012, subjective well-being has attracted wide attention around the world, encouraging the government to improve citizens’ subjective well-being through public policies [19]. Undergraduate students are one of the key objects that need to pay attention to their subjective well-being. In addition, the subjective well-being of college students is also a topic widely discussed in empirical research, and many scholars discussed subjective well-being through various ways of measurement, including one-item indicators [20,21] and multi-item indicators [22]. According to a survey of American college students, 55.9% of the students had felt helpless, 65.6% had felt lonely, and 70.8% had felt sad in the past year [4]. Some studies also mentioned that Chinese undergraduate students, affected by complex factors, generally suffered from psychological problems such as depression and anxiety [3,23]. When experiencing the transition from school to society, undergraduate students usually experience changes in their subjective well-being. Buhl (2007) compared the differences in subjective well-being between one year and four years after college graduation and identified two developing tendencies. Some graduates’ subjective well-being increased, while others gradually decreased [24]. Subjective well-being will also bring a series of influences on individual development. People with high subjective well-being tend to have higher self-efficacy, lower pressure, and more harmonious interpersonal relationships, which will jointly promote them to succeed [16,25].

Many studies have discussed the relationship between self-rated health and subjective well-being, but the direction of their relationship has always been in debate, which was mainly divided into two directions: the top-down model (subjective well-being predicts self-rated health) and the down-top model (self-rated health predicts subjective well-being) [26,27,28]. Some studies favoring the top-down model believed that subjective well-being contributed to improving health status and prolonging the lifespan [14,29]. Some studies favoring the down-top model stated that health had a significantly unidirectional impact on subjective well-being and life satisfaction [8,9,27]. Meanwhile, some studies proposed that subjective well-being and self-rated health had a bidirectional impact [30]. Moreover, previous studies mainly focused on adolescents, adults, the elderly, etc. [27,31,32,33,34]. Studies on emerging adults were mainly based on cross-sectional data and paid little attention to the prospective relationship between self-rated health and subjective well-being [35]. For instance, a national cross-sectional study in 2020 discovered that the health status of Chinese college students was significantly positively correlated with subjective well-being, and the subjective well-being decreased with increasing age [16], which reached a consistent conclusion among emerging adults in other countries [36,37]. Emerging adulthood is a transition from adolescence to adulthood, during which people usually experience the transition from college to work. During this period, health status and subjective well-being usually fluctuate [11,38]. The study of their longitudinal relationship will enrich the empirical evidence. From the perspective of practice, understanding the direction of the relationship between self-rated health and subjective well-being is helpful, providing targeted intervention to undergraduate students and prompting them to better adapt to changes in the external environment.

In general, although some studies have explored the relationship between self-rated health and the subjective well-being of undergraduate students, the prospective relationship has not attained a consensus, and the studies lack empirical data on Chinese undergraduate students. Moreover, emerging adults are at a critical transition in their lives. After students enter the labor market, the environment will change from school to society. Whether college students can make a good transition is an issue worthy of attention, and it is critical to explore the factors that can hinder the growth and development of college students. In the Chinese context, although the traditional culture of Confucianism has a profound influence on social culture, western culture has also exerted a great effect on the values of young people in recent years. Therefore, it is necessary to explore the transition of college students from university to society in the modern Chinese context, and the study complements the empirical evidence on subjective well-being and self-rated health at this significant life stage. Therefore, the study aimed to investigate the prospective relationship between self-rated health and subjective well-being in Chinese undergraduate students. According to the review, the following hypotheses are proposed:

**Hypothesis** **1.** *During the transition from college to work, the self-rated health and subjective well-being of undergraduate students present a decreasing trend;*

**Hypothesis** **2.** *Self-rated health is positively correlated with subjective well-being among undergraduate students in China;*

**Hypothesis** **3.** *Self-rated health and subjective well-being have a prospective relationship. Self-rated health in the previous year can improve subjective well-being in the following year, and subjective well-being in the previous year can improve self-rated health in the following year.*

## 2. Materials and Methods

### 2.1. Participants

The study used data collected from 2009 to 2013. Based on the probability proportional to sample size, the survey investigated a group of college students for five consecutive years, including two years in college and three years after graduation, and collected information including self-rated health and subjective well-being in the five waves. The sample size is large and representative, and some studies used other data in the survey as analysis samples [39,40,41]. In the baseline survey, 2298 students participated in the survey, and the average age of the sample was 21.57 years old. Due to a certain loss of the samples in the following survey, the study used a *t*-test to analyze the sample loss in the five waves and found that the gender, subjective well-being, and self-rated health of the lost samples were not significantly different from those of the participants (*p* > 0.05), which indicated that the sample loss was random. A total of 1021 students (537 males and 484 females) who participated in the five consecutive surveys were included in the empirical analysis.

### 2.2. Measures

#### 2.2.1. Self-Rated Health

In the questionnaire, participants were asked to respond to the question “How do you feel about your health?” to assess their current health status in the five waves, with higher scores representing better self-rated health. The full score was 100.

#### 2.2.2. Subjective Well-Being

Subjective well-being was measured by answering the question “How do you feel about your happiness?” in the questionnaire. Participants were asked to rate their subjective well-being in five waves, and the full score was 100. Higher scores implied higher subjective well-being.

### 2.3. Data Analysis

First, descriptive statistical and correlation analyses were conducted for self-rated health and subjective well-being. Second, a five-wave random intercept cross-lagged panel model (RI-CLPM) was constructed to analyze the prospective relationship between self-rated health and subjective well-being over five years. The cross-lagged panel model (CLPM) cannot distinguish the effects between and within people, and individuals vary around the same mean value of the variable. However, in RI-CLPM, individuals can vary around their own intercepts [42,43].

## 3. Results

### 3.1. Descriptive Statistics

The descriptive statistics in Table 1 include the mean (M), standard deviation (SD), minimum, and maximum of self-rated health and subjective well-being over five years. The average scores of subjective well-being increased from 83.444 (SD = 11.627) in the first year to 84.090 (SD = 11.284) in the second year (*p* = 0.249) and decreased to 77.707 (SD = 15.909) in the third year (*p* < 0.001). In the fourth year (M = 79.004; SD = 13.363; *p* = 0.017) and the fifth year (M = 80.310; SD = 12.445; *p* = 0.164), the average scores presented an increasing trend. Likewise, the average self-rated health scores increased from 80.432 (SD = 11.116) in the first year to 82.678 (SD = 9.738) in the second year (*p* < 0.001), dropped to 78.162 (SD = 12.451) in the third year (*p* < 0.001), and increased in the fourth year (M = 78.966, SD = 10.340, *p* = 0.038) and the fifth year (M = 78.937, SD = 10.641, *p* = 0.160). In general, the development of both self-rated health and subjective well-being showed an increasing-decreasing-increasing trend during two years in college and three years after graduation, but the increase was not significant. The results confirmed Hypothesis 1, i.e., that the average scores of self-rated health and subjective well-being may decline from the senior year of college to the year after graduation, which is a cause for serious concern.

### 3.2. Correlation Analysis

Table 2 shows the correlation coefficient between self-rated health and the subjective well-being of emerging adults. During two years in college and three years after graduation, the self-rated health and subjective well-being were significantly positively correlated (*p* < 0.01). Furthermore, self-rated health in the adjacent two years were significantly positively correlated (*p* < 0.01), and the scores of subjective well-being in the adjacent two years were also significantly positively correlated (*p* < 0.01). The results confirmed Hypothesis 2; that is, that the positive correlation between self-rated health and subjective well-being of emerging adults remained significant from college to post-graduation.

### 3.3. Cross-Lagged Panel Model with Random Intercepts

**Figure 1:**
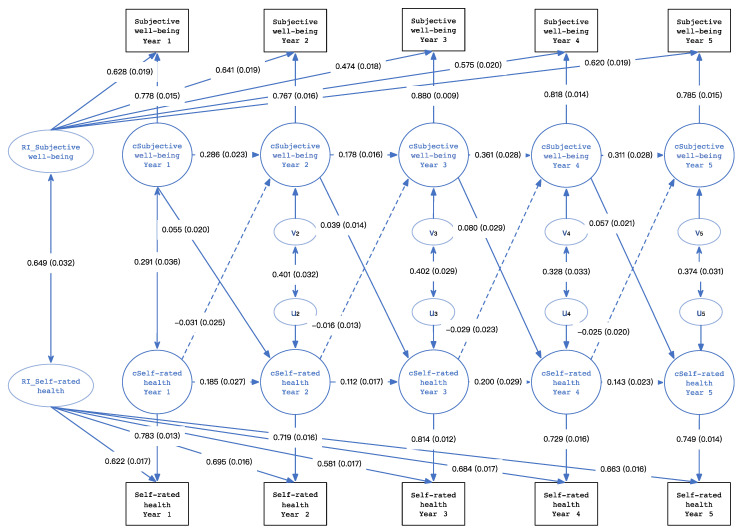
A five-wave random intercept cross-lagged panel model.

As shown in Figure 1, the study used a five-wave RI-CLPM to analyze the prospective relationship between self-rated health and subjective well-being during the transition from college to work. RI_self-rated health represented the random intercept of self-rated health, and RI_subjective well-being represented the random intercept of subjective well-being. The fit statistics of the model are good, with root mean square error of approximation (RMSEA) = 0.045; 90% CI = (0.035,0.055); CFI = 0.983, TLI = 0.977; and standardized root mean square residual (SRMR) = 0.076.

In Figure 1, the solid line indicates that the relationship between self-rated health and subjective well-being is significant, while the dotted line indicates that the relationship is insignificant. The results revealed that during two years in college and three years after graduation, subjective well-being in the previous year positively predicted subjective well-being in the subsequent year, with autoregressive standardized path coefficients ranging from 0.178 to 0.361 (*p* < 0.05). Self-rated health in the previous year positively predicted self-rated health in the subsequent year, and autoregressive standardized path coefficients ranged from 0.112 to 0.200 (*p* < 0.05). After controlling the autoregressive effects, subjective well-being in the first year positively predicted self-rated health in the second year (standardized path coefficient = 0.055, *p* < 0.05); subjective well-being in the second year positively predicted self-rated health in the third year (standardized path coefficient = 0.039, *p* < 0.05); subjective well-being in the third year positively predicted self-rated health in the fourth year (standardized path coefficient = 0.080, *p* < 0.05); and subjective well-being in the fourth year positively predicted self-rated health in the fifth year (standardized path coefficient = 0.057, *p* < 0.05). However, the cross-lagged effect of self-rated health on subjective well-being was not significant. The results partially supported Hypothesis 3; that is, that subjective well-being was a unidirectional predictor of subsequent self-rated health.

## 4. Discussion

It is believed that self-rated health and subjective well-being are positively correlated, but the longitudinal relationship in previous studies is still unclear. Based on a five-year survey during two years in college and three years after graduation, this study analyzed the prospective relationship between self-rated health and subjective well-being among undergraduate students, providing new empirical evidence and promoting society to pay more attention to undergraduate students.

Descriptive statistics showed that both self-rated health and subjective well-being significantly decreased from the senior year of college to the year after graduation, verifying Hypothesis 1. College graduation is a time of transition from school to society for most emerging adults and a time with fluctuations in physical and mental health [24]. The finding also confirms the negative impact of entering the labor market on physical and mental health [44]. However, as time goes on, their condition will gradually improve. In addition, in the Chinese context, some studies noticed that most emerging adults were ambivalent about their adult status [45]. The gap between traditional markers of adulthood and their self-perceived adulthood may also lead to a decrease in subjective well-being and self-rated health during this period. During this transition period, both physical and emotional changes often take place; thus, attention should be paid to the health status of emerging adults, and timely measures are required [46]. For instance, universities should pay more attention to the physical and mental health of senior students, carry out activities about counseling the employment pressure of senior students, and help students adapt to society better. In addition, companies should also focus on the mental health of new employees and help them adjust to work more quickly. Bauldry et al. (2012) discovered that the transition to adulthood was a healthier period compared with teenagers and young adults [47], but the results in the study indicated that emerging adults did not always maintain high health status, and similar findings have been obtained in previous studies [13]. Correlation analysis revealed that self-rated health was positively correlated with subjective well-being in the five years, which was consistent with the results of previous studies and verified Hypothesis 2 [14,15,16]. In other words, when emerging adults face the transition of graduation, their health can improve if some measures are taken to enhance their subjective well-being. Increasing health status will also benefit subjective well-being. Therefore, due to the significance of emerging adulthood and fluctuations in self-rated health and subjective well-being, some measures should be taken to improve this relationship [16].

The longitudinal relationship between self-rated health and subjective well-being has been discussed. In the cross-lagged model, the results showed that subjective well-being had a unidirectional relationship with self-rated health, which indicated that subjective well-being in the previous year unidirectionally predicted self-rated health in the following year. Hypothesis 3 is partially supported. In addition, as the cross-lagged model could explain the longitudinal relationship between two variables, the finding supported the top-down model. Higher subjective well-being can improve self-rated health in the following year, and lower subjective well-being will lead to worse self-rated health in the following year, which is consistent with previous studies [14,29]. With respect to the interpretation of the prediction, some studies noticed that the amygdala in the brain played a mediating role in the relationship between emotion and health [48]. That is, a positive effect subtly promotes health status through gray matter in the amygdala. At the theoretical level, the results have significant contributions to the longitudinal relationship between self-rated health and subjective well-being. In the relationship between the two, subjective well-being may have a greater impact on health status. From the perspective of practice, increasing subjective well-being promotes to improving one’s health [49], and senior students who are usually faced with the pressure of graduation face urgency in improving their subjective well-being to attain good health status. In addition, the study did not observe the prospective effect of self-rated health on subjective well-being. Although some findings believed that self-rated health could predict subsequent subjective well-being [8,9,27], they were mainly conducted among adults and the elderly. Adults and the elderly are generally in poorer health than emerging adults. Conversely, emerging adults who go through the transition from high school to college and college to work tend to suffer from more emotional fluctuations [13]. Therefore, we speculate that the inconsistency of results may be related to the different characteristics of the health status and subjective well-being of the groups. Given that emerging adults are at a critical transition in their lives, and they are also a significant driving force for national economic and social development, both colleges and society should pay more attention to their health and well-being, as well as taking measures to improve their subjective well-being and maintain good health for work. While attaching importance to cultivating students’ positive emotions, schools and society should actively take measures and guard against the effects of negative emotions on students’ health and growth as well [50,51]. Apart from college students, emerging adults who do not receive higher education should also be paid more attention; few studies have discussed their health status and subjective well-being, which are worthy of further exploration in future studies.

## 5. Limitations

The study has the following limitations. First, the survey assessed the participants’ subjective well-being and self-rated health, respectively, according to one question. Although this approach can effectively capture participants’ states, it may not be as precise as using scales. Second, the study only included emerging adults who attended college, and the subjective well-being and health status of those who did not attend college remain to be studied. Third, all the samples in the survey were college students in Beijing, and the generalization of the results to college students throughout China should be carefully considered.

## 6. Conclusions

The study longitudinally investigated the self-rated health and subjective well-being among Chinese undergraduate students during two years in college and three years after graduation, which is of great significance, supplementing the relevant literature and improving the subjective well-being and health of undergraduate students.

First, both self-rated health and subjective well-being significantly decreased from the senior year of college to the year after graduation. The average scores of self-rated health and subjective well-being were the highest in the senior year of college and the lowest in the year after graduation.

Second, self-rated health and subjective well-being consistently showed a significantly positive correlation.

Third, subjective well-being unidirectionally predicted self-rated health among undergraduate students. In other words, subjective well-being in the previous year positively predicted self-rated health in the following year, but self-rated health in the previous year could not affect subjective well-being in the following year.

## Figures and Tables

**Table 1:** Descriptive statistics of subjective well-being and self-rated health.

	Variables	Observation	Mean	Standard Deviation	Minimum	Maximum
Year 1	1. Subjective well-being	1021	83.444	11.627	5	100
	2. Self-rated health	1021	80.432	11.116	10	100
Year 2	3. Subjective well-being	1021	84.090	11.284	8	100
	4. Self-rated health	1021	82.678	9.738	9	100
Year 3	5. Subjective well-being	1021	77.707	15.909	8	100
	6. Self-rated health	1021	78.162	12.451	7	100
Year 4	7. Subjective well-being	1021	79.004	13.363	1	100
	8. Self-rated health	1021	78.966	10.340	7	100
Year 5	9. Subjective well-being	1021	80.310	12.445	5	100
	10. Self-rated health	1021	78.937	10.641	10	100

**Table 2:** Correlation analysis between subjective well-being and self-rated health.

	Variables	1	2	3	4	5	6	7	8	9	10
Year 1	1. Subjective well-being	1									
	2. Self-rated health	0.418 ***	1								
Year 2	3. Subjective well-being	0.519 ***	0.271 ***	1							
	4. Self-rated health	0.321 ***	0.522 ***	0.505 ***	1						
Year 3	5. Subjective well-being	0.371 ***	0.213 ***	0.395 ***	0.230 ***	1					
	6. Self-rated health	0.276 ***	0.440 ***	0.319 ***	0.492 ***	0.479 ***	1				
Year 4	7. Subjective well-being	0.390 ***	0.238 ***	0.418 ***	0.252 ***	0.549 ***	0.313 ***	1			
	8. Self-rated health	0.279 ***	0.440 ***	0.276 ***	0.497 ***	0.294 ***	0.551 ***	0.455 ***	1		
Year 5	9. Subjective well-being	0.353 ***	0.173 ***	0.423 ***	0.245 ***	0.452 ***	0.287 ***	0.609 ***	0.323 ***	1	
	10. Self-rated health	0.309 ***	0.373 ***	0.335 ***	0.413 ***	0.310 ***	0.471 ***	0.383 ***	0.576 ***	0.512 ***	1

Note: *** *p* < 0.01.

## Data Availability

The data that support the findings of this study are available from the corresponding author upon reasonable request.
